# Toxoplasmosis: Current and Emerging Parasite Druggable Targets

**DOI:** 10.3390/microorganisms9122531

**Published:** 2021-12-07

**Authors:** Rana El Hajj, Lina Tawk, Shaymaa Itani, Maguy Hamie, Jana Ezzeddine, Marwan El Sabban, Hiba El Hajj

**Affiliations:** 1 Department of Biological Sciences, Beirut Arab University, P.O. Box 11-5020, Riad El Solh, Beirut 1107 2809, Lebanon; r.hajj@bau.edu.lb; 2 Department of Medical Laboratory Sciences, Faculty of Health Sciences, University of Balamand, Beirut 1100 2807, Lebanon; lina.tawk@balamand.edu.lb (L.T.); jezzeddine25@gmail.com (J.E.); 3 Department of Experimental Pathology, Microbiology and Immunology, Faculty of Medicine, American University of Beirut, P.O. Box 11-0236, Riad El-Solh, Beirut 1107 2020, Lebanon; ski02@mail.aub.edu (S.I.); mh242@aub.edu.lb (M.H.); 4 Department of Anatomy, Cell Biology and Physiological Sciences, Faculty of Medicine, American University of Beirut, P.O. Box 11-0236, Riad El-Solh, Beirut 1107 2020, Lebanon; me00@aub.edu.lb

**Keywords:** acute toxoplasmosis, chronic toxoplasmosis, parasite therapeutic targets, neuropathies, antiparasitic drugs, immunomodulatory drugs

## Abstract

Toxoplasmosis is a prevalent disease affecting a wide range of hosts including approximately one-third of the human population. It is caused by the sporozoan parasite *Toxoplasma gondii* (*T. gondii*), which instigates a range of symptoms, manifesting as acute and chronic forms and varying from ocular to deleterious congenital or neuro-toxoplasmosis. Toxoplasmosis may cause serious health problems in fetuses, newborns, and immunocompromised patients. Recently, associations between toxoplasmosis and various neuropathies and different types of cancer were documented. In the veterinary sector, toxoplasmosis results in recurring abortions, leading to significant economic losses. Treatment of toxoplasmosis remains intricate and encompasses general antiparasitic and antibacterial drugs. The efficacy of these drugs is hindered by intolerance, side effects, and emergence of parasite resistance. Furthermore, all currently used drugs in the clinic target acute toxoplasmosis, with no or little effect on the chronic form. In this review, we will provide a comprehensive overview on the currently used and emergent drugs and their respective parasitic targets to combat toxoplasmosis. We will also abridge the repurposing of certain drugs, their targets, and highlight future druggable targets to enhance the therapeutic efficacy against toxoplasmosis, hence lessening its burden and potentially alleviating the complications of its associated diseases.

## 1. Introduction

*Toxoplasma gondii* (*T. gondii*) is an obligate intracellular protozoan parasite infecting a broad range of animals, including one-third of the world’s human population [1]. Because of its high prevalence in the United States, the Centers for Disease Control and Prevention classified toxoplasmosis among the neglected parasitic infections that require a public health action control [2]. The pathogenesis of *T. gondii* varies among patients. The acute form of the disease develops a few days after the infection and is asymptomatic in more than 80% of immunocompetent individuals [1,3]. The remaining patients may exhibit general flu-like symptoms, fever, myalgia, and cervical posterior adenopathy, among other symptoms [1]. In certain regions, e.g., French Guiana and Latin America, severe symptoms including fatal pneumonitis, myocarditis, meningoencephalitis, and polymyositis were noted in some immunocompetent patients who contracted atypical strains of *T. gondii* [4].

Congenital toxoplasmosis occurs in sero-negative pregnant women acquiring *T. gondii* as primary infection. The parasite crosses the blood–placenta barrier and reaches the fetus [5]. The transmission rate and severity of congenital toxoplasmosis depend on the gestational trimester at which the infection is acquired [6,7]. Transmission rates of 25% are estimated when the infection occurs during the first trimester, while 54 and 65% transmission rates are estimated when infection occurs during the second or third trimesters, respectively [7,8]. Infection of the fetus during the first trimester often leads to abortion. However, in cases of stillbirth, the baby suffers from severe aberrations of the brain (hydrocephalus, intracranial calcifications, deafness, mental retardation, seizures) and the eyes (retinochoroiditis that may lead to blindness (reviewed in [9])). Infection of the fetus during the second or third trimester is less likely to cause abortion; however, retinochoroiditis and learning difficulties may occur after birth [10]. Retinochoroiditis is the most common symptom of ocular toxoplasmosis and predominantly results from an acquired congenital toxoplasmosis. It presents with posterior uveitis, vitritis, focal necrotizing granulomatous retinitis, and reactive granulomatous choroiditis [11]. It is worth noting that the acquisition of toxoplasmosis during pregnancy varies according to regions [12,13], and atypical *T. gondii* genotypes were identified and led to re-infection of previously sero-positive pregnant women [14], resulting in a more severe congenital disease [15].

Following an acute infection, *T. gondii* targets the brain and skeletal muscles, where it persists as latent tissue cysts responsible for chronic toxoplasmosis (reviewed in [16]). The switch from the acute to the chronic form is triggered by the host immune response, among other factors (reviewed in [17,18]). Although the direct symptoms of chronic toxoplasmosis are not fully characterized in healthy individuals, chronic toxoplasmosis was regarded as clinically asymptomatic [19]. Yet, the brain immune response triggers inflammation, which disrupts neuronal connectivity and associates with ventricular dilatation [20,21,22]. In addition, chronic toxoplasmosis correlates with several neuropathies [19], behavioral disorders, and cancers [23]. Nonetheless, direct molecular proofs remain to be elucidated [24,25] (Daher et al., in press).

Immunosuppression triggers the reactivation of chronic toxoplasmosis, leading to serious complications and potential fatality [1,26,27,28]. Reactivation was mostly documented in HIV patients or patients treated with immunosuppressive therapies prior to solid organ or hematopoietic stem cell transplantation [18,29,30,31,32,33,34,35]. Indeed, among solid organ transplanted patients, orthotopic heart transplant recipients presented with the highest risk of reactivation of toxoplasmosis, owing to the high propensity of *T. gondii* cysts in striated muscles [36]. Chemotherapy administration, in particular, rituximab, also triggered the reactivation of toxoplasmosis [37,38,39,40]. HIV patients with reactivated toxoplasmosis manifest predominantly with neurological symptoms including toxoplasmic encephalitis, encephalopathy, meningoencephalitis, headache, seizures, and poor coordination, while transplanted patients exhibit a more disseminated status [4,41].

## 2. Current Treatment Modalities of Toxoplasmosis

Treatment of toxoplasmosis remains limited to general antiparasitic/antibacterial drugs (reviewed [42,43]). Therapeutic strategies for this parasitic disease vary according to the disease state and the host immune system.

In congenital toxoplasmosis, the combination of pyrimethamine and sulfadiazine is the recommended first-line therapy (Table 1) [44,45,46,47]. Pyrimethamine is an inhibitor of dihydrofolate reductase (DHFR) enzyme, known to block the synthesis of purines and pyrimidines. Sulfadiazine is an inhibitor of dihydropteroate synthase (DHPS). Since *T. gondii* synthesizes folates de novo [44], this combination exhibits its antiparasitic activity through blocking the biosynthesis of parasitic folate, thus interrupting nucleic acid synthesis and parasite replication. To reduce the harmful side effects, among which is bone marrow myelosuppression, pyrimethamine/sulfadiazine is administered with folinic acid (leucovorin), which is an active metabolite of folic acid and an essential co-enzyme for nucleic acid synthesis [42,48,49]. Yet adverse side effects still present and comprise neutropenia, thrombocytopenia, leukopenia, and teratogenic potential, if administered during the first trimester of pregnancy. In rare cases agranulocytosis, toxic epidermal necrolysis, and hepatic necrosis may also manifest [50,51,52] (Table 1). In pregnant women suspected to have toxoplasmosis, spiramycin is given prophylactically (reviewed in [43,47]) (Table 1). Spiramycin is a macrolide antibiotic that accumulates in and does not cross the placental barrier, hence preventing the materno–fetal transmission of the parasite. Nonetheless, when the diagnosis confirms the infection of the fetus or neonate, spiramycin treatment should be withdrawn and replaced by the conventional treatment pyrimethamine/sulfadiazine/leucovorin [53].

In immunocompetent patients, acute toxoplasmosis is generally asymptomatic; hence, it does not require any therapeutic intervention. Nevertheless, it was reported in immunocompetent patients of certain areas including South America that atypical strains caused severe complications with multivisceral involvement and potentially life-threatening outcome [54,55]. These symptomatic patients, whether presenting with severe symptoms or following ocular involvement or, even if very rare, following a laboratory-acquired infection, are treated with pyrimethamine/sulfadiazine/folinic acid, pyrimethamine/clindamycin/folinic acid, pyrimethamine/folinic acid/atovaquone, pyrimethamine/azithromycin/folinic acid or trimethoprim/sulfamethoxazole (Table 1) (reviewed in [42]). Of note, clindamycin and azithromycin are antibiotics targeting protein synthesis while atovaquone is a quinone antimicrobial medication targeting the mitochondrial electron transport and the mitochondrial cytochrome bc1 complex (Table 1). Attempts to combine pyrimethamine with atovaquone, clindamycin, or azithromycin proved less effective than pyrimethamine/sulfadiazine. The same results were obtained using cotrimoxazole (trimethoprim-sulfamethoxazole) or atovaquone monotherapy [42,56]. The aforementioned treatment modalities spare chronic toxoplasmosis and target only the acute form of the infection [48,57,58,59]. Reactivation of tissue cysts, which are the hallmark of chronic toxoplasmosis, occurs when the host immunity is suppressed [18,29,30,31,32,33,34]. For instance, in HIV patients an association between CD4 counts and *T. gondii*-related neurologic symptoms was reported [27]. Reactivation becomes a concern if CD4 counts fall below 200 cells/µL and is the consequence of decreased IFN-γ and cytokine production. In these patients, toxoplasmic encephalitis is the major clinical manifestation and leads to fatality if left untreated [60]. Symptomatic HIV patients revealing fever and dizziness as part of their *Toxoplasma* encephalitis prodrome pursued medical care quicker than those who did not present with these symptoms, leading to rapid therapeutic intervention, hence reduced rates of mortality [61]. Induction treatment with pyrimethamine/sulfadiazine resulted in 80% response rates [62]. However, severe adverse effects were encountered ranging from fever to skin rash to hematologic complications (leukopenia and thrombocytopenia). Thus, leucovorin was used to overcome these side effects (Table 1) [62]. Atovaquone monotherapy was not effective; nevertheless, induction therapy using the combination of sulfadiazine/pyrimethamine/atovaquone led to high response rates (reviewed in [63]). Anti-retroviral therapy (ART) increased the survival and decreased the mortality and relapse rates in HIV patients with opportunistic infections including reactivation of toxoplasmosis [64,65,66,67,68,69]. In transplant patients, disseminated toxoplasmosis occurs frequently [4,41]; therefore, prophylaxis or even empirical initiation of treatment is recommended in suspected cases and before confirming diagnosis. In a retrospective study, it was demonstrated that the combination of sulfadiazine/pyrimethamine marginally improved survival in hematopoietic stem cell transplantation [70]. In solid organ transplant recipients, where reactivation of chronic toxoplasmosis occurs either as a manifestation derived from an infected donor or occasionally as a primary acquired infection following transplantation, a standard and effective treatment is still lacking, and the therapeutic modalities that are adopted are similar to those used in HIV patients (Table 1) (reviewed in [42,43]).

## 3. Drug Resistance in *Toxoplasma gondii* Infections

A gold standard treatment for toxoplasmosis is still lacking. In addition to the multiple reported side effects of clinically used drugs, the emergence of resistance strains was described [48,58,71,72]. This resistance may be one of the factors dictating the failure of treatment in patients with acute toxoplasmosis or in patients who relapse in the course of suppressive therapy. However, the extent at which this drug resistance leads to the failure of treatment cannot be determined easily because of the complex recovery of *T. gondii* from infected patients [48]. Nonetheless, alterations in the common enzyme targets of the folate pathway, *dhfr* and *dhps,* were described [73,74,75,76]. In that sense, in a sulfadiazine-resistant Brazilian isolate from newborns with congenital toxoplasmosis, a large number of polymorphisms were identified in the *dhps* gene. Yet, no association was found between the profile of susceptibility to sulfadiazine and any identified *dhps* variants [77]. Three clinically isolated strains (the atypical RMS-2001-MAU strain, the type I B1 strain, and the Type II RMS-1995-ABE strain) were also shown to be resistant to sulfadiazine [78]. Using a proteomic approach, 31 proteins were differentially modulated between sulfadiazine-sensitive and resistant strains of *T. gondii,* of which the rhoptry protein ROP2A virulence factor was highly abundant in two naturally resistant Type II strains, TgH32045 and TgH32006 [79]. To overcome these problems, continuous efforts to develop promising drug candidates are being made [59,80].

## 4. Emerging Therapeutic Targets in *Toxoplasma gondii* Infections

**Figure 1:**
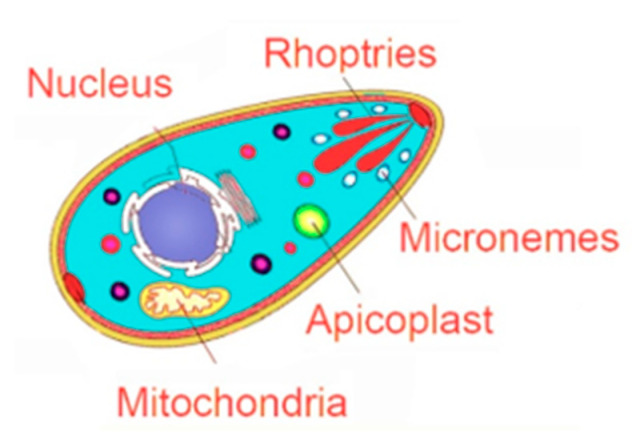
Schematic representation of known *Toxoplasma gondii* therapeutic targets.

Despite all prophylactic approaches to prevent the infection with *T. gondii*, an available human vaccine is still out of reach. While the search for a vaccine has been highly pursued, currently used drugs target only the acute form of the disease. Chronic toxoplasmosis, which represents the more prevalent form and associates with dreadful clinical outcomes, reaching fatality in immunocompromised patients, remains an unmet medical need. An ideal drug against toxoplasmosis should affect multiple stages of the parasite life cycle (i.e., tachyzoites responsible for acute toxoplasmosis and bradyzoites responsible for chronic toxoplasmosis). Furthermore, these drugs should (1) target the parasite biology, (2) exhibit low toxicity and tolerable side effects, (3) have high bioavailability, and (4) cross the blood–brain barrier and reach the brain, where the propensity for neuronal cysts is high [81]. A number of preclinical studies were conducted in vitro and prolonged mice survival in vivo (reviewed in [82]). A comprehensive summary of tested drugs and compounds over a decade extending from 2006 to 2016 reported 80 clinically available drugs and a large number of new compounds with more than 40 mechanisms of action. Several target-based drug screens were also identified. These include different kinases, mitochondrial electron transport chain, fatty acid synthesis, DNA synthesis, and replication, among several others [59]. In the following sections, we will provide a comprehensive overview of the different parasite targets and their corresponding emerging drugs (Figure 1, Table 2).

### 4.1. Targeting the Apicoplast

In apicomplexan parasites, the apicoplast is a nonphotosynthetic organelle formed following endosymbiosis of a green algae. This organelle assumes several functions including the biosynthesis of fatty acids, lipoic acid, and isoprenoids, among other metabolites [83]. The absence of apicoplasts in mammalian cells made them an excellent therapeutic target, and several attempts to target their parasitic functions have been tested (Figure 1, Table 2). These therapeutic targets span several apicoplast enzymes involved in fatty acid synthesis and metabolism. These include the fatty acid synthase II (FASII) [84], the acetyl CoA carboxylase (ACC) that catalyzes the formation of Malonyl-CoA, and the β-ketoacyl ACP synthase III (FabH) [85,86,87]. Clodinafop, thiolactomycin, and triclosan proved efficient in targeting these enzymes and blocking the parasite fatty acid synthesis [85,86].

Another apicoplast therapeutic drug target is the isoprenoid synthesis pathway. Isoprenoid is a precursor of ubiquinone and sterols, playing an important role in cell signaling. 1-deoxy-D-xylulose-5-phosphate (DOXP) was identified in the apicoplast and plays a role in the biosynthesis of isoprenoid [88]. Two key enzymes featured in the DOXP pathway, the DXP reducto-isomerase and DXP synthase, were identified in apicomplexan parasites including *T. gondii* and are absent in humans, posturing these enzymes as attractive therapeutic drug targets [88]. Fosmidomycin antibiotic proved efficient against these two enzymes; however, resistance problems, high concentrations, and low bioavailability hindered its activity [89,90].

Because of its prokaryotic nature, the apicoplast’s DNA is circular, and its unwinding and supercoiling during replication is controlled and mediated by DNA topo-isomerases including the DNA gyrase [91,92,93]. This enzyme is absent in humans and was the target of several quinolone and fuoroquinolone antibiotics. These include ciprofloxacin, trovafloxacin, ofloxacin and temafloxacin, which exhibited in vitro and in vivo efficacy against *T. gondii* infections [94,95]. In addition to DNA replication, the prokaryotic nature of the apicoplast also confers a mechanism of protein synthesis independent of that occurring in the nucleus of the parasite. Indeed, the apicoplast encodes proteins and RNA indispensable to its ribosomes, hence its specific proteins [96]. Clindamycin, spiramycin, and azithromycin, known to affect prokaryotic protein synthesis, exhibited toxoplasmicidal activities in vitro and in vivo [97].

### 4.2. Targeting the Invasion Complex

#### 4.2.1. Microneme Organelles

Micronemes are small rod-shaped organelles of the apical complex of *T. gondii.* They play a chief role in attachment, gliding, motility, and egress during the invasion process required for parasite survival [98,99,100]. Owing to their uniqueness in apicomplexan parasites, their absence in mammalian cells, and their pivotal role in invasion, micronemes and especially their calcium-mediated secretion have been at the core of parasite targeting. In that sense, *T. gondii* calcium-dependent protein kinase 1 (TgCDPK1), a parasite cytosolic serine/threonine-protein kinase regulating the calcium-dependent pathway, is essential for micronemal protein secretion. The inhibition of this enzyme impairs host cell invasion capacity [101,102,103]. Several bumped kinase inhibitors (BKIs) selectively inhibited TgCDPK1 [104,105] (Figure 1, Table 2). BKI-1294, a pyrazolo-pyrimidine-based compound, resulted in high inhibition of invasion in vitro and high efficiency against acute toxoplasmosis in vivo when given orally [105]. This same inhibitor also proved efficient against congenital toxoplasmosis in a murine model [106]. The promising efficacy of BKI-1294 was hindered by its cardiac toxicity, halting its clinical development [107,108,109]. Consequently, BKI-1294 was chemically modified by maintaining its TgCDPK1 selectivity and efficacy and reducing its cardiac toxicity. Compound 32 was thus developed and proved efficacious in vitro and in vivo, in particular, through reducing brain cysts [108]. Other pyrazolo-pyrimidine inhibitors of TgCDPK1 were also tested. Another compound (called compound 24) exhibited in vitro nanomolar and submicromolar activity on TgCDPK1 and inhibited parasite proliferation [110]. This BKI analog showed an excellent oral bioavailability, reduced acute and chronic toxoplasmosis in mice, and, more importantly, delayed reactivation of the chronic disease following immunosuppression [110]. Recently, BKI-1748, a 5-aminopyrazole-4-carboxamide compound inhibited proliferation of *Neospora caninum* and *Toxoplasma gondii* in vitro. The safety of this compound was tested in zebrafish with no embryonic impairment up to 10 μM, and in pregnant mice, with no pregnancy interference at a dose of 20 mg/kg/day. The efficacy of BKI-1748 was assessed in standardized pregnant mouse models infected with tachyzoites or oocysts of *T. gondii* and resulted in increased pup survival and profound inhibition of vertical transmission [111]. SP230, an imidazo[1,2-b]pyridazine salt targeting TgCDPK1, proved efficient against murine acute toxoplasmosis in mice. Importantly, administration of SP230 yielded significant efficacy against congenital toxoplasmosis. SP230 resulted in the reduction of parasite burden in 97% of fetuses [112].

#### 4.2.2. Rhoptry Organelles

Rhoptries are organelles of the apical complex discharging their content during parasite invasion. Their protein content plays several roles including contributing to the formation of moving junction and the parasitophorous vacuole membrane (PVM). Furthermore, other functions were unraveled, and some rhoptry proteins play roles as virulence factors, others manipulate host signaling, while others play a role in immune evasion [113,114,115].

ROP2 family contains a group of proteins, with some members sharing more than 70% identity, while other members are structurally more divergent [116]. While the members of this family evolved with all the elements to be active kinases, some members (ROP2, ROP4, ROP7, ROP5) lost some key motifs or residues in the kinase activity domain over time to acquire other functions [116,117]. For instance, ROP2 contributes [118], but is not the only factor [119], to the recruitment of the host mitochondria around the PVM. ROP5 exhibits an inverted topology in the PVM as compared to other members of the family [120], and protein forms a complex with ROP17 and ROP18 (which retained their kinase activity), hence controlling the virulence in mice [121,122]. In that sense, ROP5 and ROP18 allele combinations are tightly related to *T. gondii* virulence [122,123,124,125], and ROP5 teams up with ROP18 and complements its activity to inhibit the accumulation of the IFN-γ-induced immunity-related GTPases (IRGs) in vivo, hence contributing to the pathogenesis and immune evasion [126]. Owing to the role of ROP5 and ROP18 in virulence, attempts to use this complex as a vaccine strategy were promising in mice [127]. In addition, recombinant ROP5 and ROP18 were evaluated for their diagnostic potential in human toxoplasmosis [128]. ROP16 and ROP18 were also proven as virulence factors through targeting the host cell nucleus and exhibiting their kinase activity to phosphorylate key proteins involved in cell cycle and different signaling pathways [129]. ROP18 is expressed in genotypes I/II demonstrating their role in controlling the virulence of the parasite [130], and transfection of the virulent ROP18 allele into a nonpathogenic type III strain confers virulence and enhances mortality in vivo [131]. Through its kinase activity, ROP18 phosphorylates GTPases, promoting macrophage survival and virulence [132] and ensuring an immune evasion strategy for virulent strains [133]. ROP16, on the other hand, is expressed in genotypes I/III and also plays a key role in the virulence of the parasite [130]. ROP16 phosphorylates STAT3 and STAT6 [134], hence downregulating IL-12, which plays a chief role in mounting an immune response against *T. gondii* infection [130]. ROP16 also suppresses T cell activity, hence ensuring immune cell evasion [135]. Moreover, direct phosphorylation of STAT3 by ROP16 mimics the IL-10 activity and downregulates IFN-γ, hence enhancing the virulence of *T. gondii* [134]. Recently, ROP16-mediated activation of STAT6 proved important for type III *T. gondii* survival through suppression of host cell reactive oxygen species production [136]. Moreover, ROP16 kinase activity silences the *cyclin B1* gene promoter, hijacking the function of the host cell epigenetic machinery [137]. The role of ROP proteins in the virulence of the parasite makes them excellent drug target candidates to combat toxoplasmosis. A high-throughput screen to identify small molecule inhibitors of ROP18 identified several inhibitors belonging to oxindoles, 6-azaquinazolines, and pyrazolopyridines chemical scaffolds. Treatment of IFN-γ-activated cells with one of these inhibitors enhanced immunity-related GTPase recruitment to wild type parasites [138]. Thiazolidinone derivatives inhibited *T. gondii* in vitro, and in silico analysis demonstrated that the best binding affinity of these derivatives was observed in the active site of kinase proteins with a possible effect of one derivative in the active site of ROP18 [139] (Figure 1, Table 2).

### 4.3. Targeting the Parasite Mitochondrial Electron Transport Pathway

In apicomplexan parasites, the mitochondrial electron transport chain is of central importance for energy production [140]. This complex, present in the mitochondrial electron transport chain, was targeted by several mitochondrial inhibitors, hindering cell respiration and leading to parasite death (Figure 1, Table 2). Atovaquone, clinically used in the treatment and prophylaxis of toxoplasmosis, is an inhibitor of the hydroquinone oxidation site of the bc1 complex [48]. Emerging resistance of the parasite limited its use [141]. Different quinolone derivatives including the endochin-like quinolones (ELQs), which target the hydroquinone reduction site of bc1, have been developed. ELQ-271 and ELQ-316 inhibited parasite growth at nanomolar concentrations in vitro and reduced the number of brain cysts in murine models [142,143,144]. Another compound, ELQ-400, alleviated the burden of acute toxoplasmosis in mice and demonstrated 100% cure rates upon infection of mice with a type I lethal strain [143,145].

Naphthoquinones bind to the hydroquinone oxidation site of the bc1 complex. Seven naphthoquinones exhibited an anti-*T. gondii* inhibitory effect in vitro. Three out of seven (para-hydroxynaphthoquinones) were able to enhance survival of mice following infection with a virulent *T. gondii* strain (Ferreira et al., 2002) (Figure 1, Table 2).

### 4.4. Targeting the Interconversion between Tachyzoites and Bradyzoites

Histone acetylase (HAT) and histone deacetylase (HDAC) enzymes controlling histone acetylation regulate and control the parasite gene expression during the back and forth interconversion between acute and chronic toxoplasmosis. Targeting these enzymes is a plausible therapeutic scenario. The cyclopeptide FR235222, a TgHDAC3 inhibitor, induced in vitro conversion to bradyzoites and inhibited parasite growth [146]. To decrease the toxicity of FR235222, W363 and W399 derivatives were generated and exhibited equivalent IC50 to the mother compound in vitro [147].

Rolipram, a phosphodiestrase-4 (PDE4) inhibitor interfered with the interconversion from tachyzoites to bradyzoites through immunomodulatory activities and significantly reduced the cyst burden in the brains of chronically infected mice [148]. Guanabenz, an FDA-approved antihypertensive drug, interferes with translational control in tachyzoite and bradyzoite stages through inhibition of dephosphorylation of *T. gondii* eukaryotic initiation factor 2 (TgeIF2). This inhibitor protected mice against acute toxoplasmosis and reduced the brain cyst numbers in chronically infected mice [81].

*T. gondii* mitogen-activated protein kinase (MAPK) regulates parasite proliferation, response to stress, and stage differentiation. Pyridinylimidazole inhibited TgMAPK1, caused morphological changes, and reduced the virulence of *T. gondii* [149,150,151]. In conclusion, targeting the interconversion between tachyzoites and bradyzoites can be a promising therapeutic approach.

Heat shock proteins (HSPs) promote host cell invasion, parasite growth, survival, as well as stage conversion from tachyzoite to bradyzoite, hence from the acute to the chronic form of infection [152,153]. HSP60 and 70 are important in the development and survival of *T. gondii*. While HSP60 is responsible for stage-specific induction of the respiratory pathway, HSP70 plays a role in stage differentiation and virulence [154]. HSP70 protects the parasite from the host immune system. Treatment of mice with quercetin and oligonucleotide reduced HSP70 expression in a virulent *T. gondii* strain [155]. The 3D structures for *T. gondii* Hsp60 and Hsp70 were performed by homology modeling, and a virtual screening of 1560 compounds from the NCI Diversity Set III was analyzed and demonstrated that the major exhibited interactions were hydrogen bonding and hydrophobic interactions in binding to HSP60 and HSP70, providing guidelines for the development of inhibitors for these parasitic heat shock proteins [156].

## 5. Drug Repositioning: A Promising Approach against *T. Gondii*

Drug repositioning became an advantageous approach to explore more clinical usages of existing drugs. This strategy offers multiple advantages, including the established pharmacokinetic and pharmacodynamic properties of drugs, their assumed targets, reduced cost and timeline, hence accelerating their clinical applications.

Since *T. gondii* and *Plasmodia* spp. belong to the phylum Apicomplexa, several anti-malarial drugs were tested against *T. gondii*. The piperazine acetamide MMV007791, a potent anti-malarial drug, demonstrated the highest efficacy and selectivity against *T. gondii* [157]. This drug, along with six out of 400 blood stage-active anti-*Plasmodium* compounds, was screened from the open access Medicines for Malaria Venture Malaria Box and exhibited toxoplasmicidal efficacy [157]. A screen of a wider open access library of 400 compounds, referred to as the Pathogen box, in which several drugs tested pre-clinically against other apicomplexan parasites including *Crysptosporidium parvum* and *Neopsora caninum*, revealed the efficacy of 18 compounds against *T. gondii* [158]. Among these, the anti-*Neospora caninum* buparvaquone and the anti-*Crysptosporidium parvum* MMV675968 targeting the mitochondrial electron transport and DHFR, respectively, exhibited a similar targeted activity in *T. gondii* [158]. In another screen of a chemical compound library by the Drug Discovery Initiative at Japan, two inhibitors, tanshinone IIA (with potential anti-cancer activity) and hydroxyzine (a first-generation antihistamine drug), were identified and seemed to target intermediately differentiated bradyzoites [159].

Tetraoxane, an anti-cancer molecule, significantly prolonged survival of acutely infected mice as compared to control mice, highlighting a potential use of this molecule against toxoplasmosis [160]. Miltefosine, extensively used currently in the treatment of visceral leishmaniasis among other protozoal infections, was not efficient against acute toxoplasmosis but reduced brain cyst burden in chronically infected mice [161]. More recently, the ability of a collection of 666 FDA-approved compounds (Selleck New Compound Library) to inhibit *Toxoplasma* growth was screened. A total of 68 compounds proved effective and inhibited parasite growth, out of which two compounds, NVP-AEW541 and GSK-J4 HCl, inhibited tachyzoite invasion and proliferation by halting cell cycle progression from G1 to S phase, respectively. Both compounds prolonged survival of acutely infected mice with *T. gondii* and remarkedly reduced the parasite burden of tissues [162]. Another screen encompassed the ability of 1120 compounds to reduce *Toxoplasma* replication. A total of 94 compounds, including inhibitors of dopamine or estrogen signaling, blocked parasite replication. Tamoxifen, an established inhibitor of the estrogen receptor, also reduced parasite invasion and replication [163].

Furthermore, a broad spectrum of known DNA damage inducers were evaluated as anti-toxoplasmic drug targets (reviewed in [164]). These genotoxic drugs include topoisomerase-2 inhibitors such as daunorubicin [165], trovafloxacin [95], enrofloxacin [166], and gatifloxacin [167], which proved effective against *T. gondii*. Topoisomerase-1 inhibitors, including artemisinin, a highly potent anti-malarial dug, and several of its derivatives, mostly artemisone and artemiside, reduced brain cyst burden in chronically infected mice [48,168] and prolonged survival of mice following reactivation of toxoplasmosis [169]. Artemether, with similar topoisomerase-1 inhibitory activities, also exhibited toxoplasmicidal activities [168,170,171]. Moreover, other topoisomerase-1 inhibitors including harmane, harmine, and nor-harmane Top1 inhibitors proved efficient against *T. gondii* [48]. DNA-intercalating agents including fluphenazine, thioridazine, trifluoperazine, hycantone [159,172], phleomycin [173], mitomycin C [165], the ribonucleotide reductase inhibitors thiosemicarbazones and hydroxyurea [174], the oxidative DNA damage/DNA binding resveratrol [165], and valproic acid [175] also exhibited an anti-*Toxoplasma* activity (Figure 1, Table 2).

The incessant advances and the enhanced understanding of the immunopathogenesis of toxoplasmosis gave new insights into counteracting immune damages. Hence, immunomodulation strategies have proved efficient to combat acute and, more importantly, chronic toxoplasmosis. In that sense, imiquimod, an FDA-approved immune-modulatory drug for topical use against some viral infections [176] and against cutaneous leishmaniasis [176,177,178,179,180], was explored in murine models of acute and chronic toxoplasmosis. During acute toxoplasmosis, imiquimod treatment reduced the brain cyst burden and impaired the infectivity of the remaining ones. More importantly, treatment of chronically infected mice with imiquimod significantly abridged the number of brain cysts and led to delayed or abortion of reactivation upon immunosuppression [181]. At the molecular level, imiquimod upregulated the expression of toll-like receptors and activated the MyD88 pathway, resulting in the induction of the immune response to control reactivation [181]. Ursolic acid, a natural pentacyclic triterpenoid compound [182], inhibited *T. gondii* survival, downregulated several proteins of the apical complex including ROP18 and MIC8, and augmented the production of NO, ROS, IL-10, IL-12, GM-CSF, and IFN-γ, while reducing the expression of IL-1, IL-6, TNF-α, TGF-βin *T. gondii*-infected immune cells, highlighting the immune-modulatory activities of this natural compound [183,184].

## 6. Concluding Remarks

Despite its prevalence, toxoplasmosis remains a neglected disease of human and veterinary importance. Clinically available drugs target acute but not chronic toxoplasmosis. These drugs are associated with a spectrum of adverse side effects. Moreover, emergence of resistance in parasites was documented. Identifying new drug targets is paramount in creating avenues for new treatment modalities to overcome the drawbacks of these clinically used drugs and improve their pharmacokinetics, bioavailability in target organs (eyes, placenta, fetal compartment, etc.), and, more importantly, their access to the brain after crossing the blood–brain barrier. It is worth noting that chronic toxoplasmosis resides in the brain and skeletal muscles and is associated with severe complications in immunocompromised patients. Ideally, drug candidates should be cost-effective and should act on acute replicating tachyzoite and latent bradyzoite stages, hence preventing acute disease (including newly acquired infection, ocular and congenital toxoplasmosis) and allowing the resolution of chronic toxoplasmosis, its associated diseases, and, more prominently, its reactivation. Several drug targets including unique organelles of the parasite (apicoplast, apical complex organelles (rhoptries and micronemes)) were identified. Drug repurposing was also applied and deemed a useful approach to combat toxoplasmosis. Under this category fall several anti-malarial drugs, anti-cancer and genotoxic drugs, and, more importantly, immunomodulatory drugs that activate the immune response, triggering a toxoplasmicidal outcome. Recent advances in high-throughput sequencing, modeling, and proteomics techniques should help in identifying parasite markers that do not target host factors, hence increasing their specificity and lessening their toxicity. There is also a surge in developing quick diagnostic tests to augment the clinicians’ awareness of reactivation in immunocompromised patients. Finally, because of the importance of the host immune system in controlling the persistence and the back and forth switch between acute and chronic toxoplasmosis, targeting the parasite and enhancing the host immune system against it should be considered as simultaneous approaches to eradicate different forms of the infection. This urgency to develop effective drugs against toxoplasmosis has gained a new dimension following the plethora of research avenues that have associated this infection with major primary neuropathies, behavioral and psychiatric disorders, and some cancers.

## Figures and Tables

**Table 1:** Therapeutic modalities of toxoplasmosis.

Toxoplasmosis	Currently Used Drugs	Mechanism(s) of Action
**Congenital toxoplasmosis**		
Maternal congenital toxoplasmosis or confirmed infection of neonate or fetus following congenital toxoplasmosis	^1^ Pyrimethamine (Inhibitor of dihydrofolate reductase (DHFR))	Inhibition of the biosynthesis of parasitic folate, interrupting nucleic acid synthesis and parasite replication
	+ ^1^ Sulfadiazine (Inhibitor of dihydropteroate synthase)	
	+ Folinic acid (leucovorin)	Reduction of the harmful side effects (i.e., bone marrow myelosuppression)
Suspected congenital toxoplasmosis	Spiramycin	Inhibition of protein synthesis
**Toxoplasmosis in immunocompetent patients**		
Acute toxoplasmosis	^2^ Pyrimethamine (Inhibitor of dihydrofolate reductase (DHFR))	Inhibition of the biosynthesis of parasitic folate, interrupting nucleic acid synthesis and parasite replication
	+ ^2^ Sulfadiazine (Inhibitor of dihydropteroate synthase)	
	+ Folinic acid (leucovorin)	
		Reduction of the harmful side effects (i.e., bone marrow myelosuppression)
	^2^ Pyrimethamine	Inhibition of the biosynthesis of parasitic folate
	+^3^ Clindamycin	Inhibition of protein synthesis
	+ Folinic acid	Reduction of the harmful side effects (i.e., bone marrow myelosuppression)
	^2^ Pyrimethamine	Inhibition of the biosynthesis of parasitic folate
	+ Folinic acid	Reduction of the harmful side effects (i.e., bone marrow myelosuppression)
	+ Atovaquone	Targeting the mitochondrial electron transport and the mitochondrial cytochrome bc1 complex
	^2^ Pyrimethamine	Inhibition of the biosynthesis of parasitic folate
		Reduction of the harmful side effects (i.e., bone marrow myelosuppression)
	+ Folinic acid	
		Inhibition of protein synthesis
	+ ^4^ Azithromycin	
	^2^ Trimethoprim	Inhibition of the biosynthesis of parasitic folate
	+ Sulfamethoxazole	Bacteriostatic sulfonamide interfering with folic acid synthesis
**Ocular toxoplasmosis**		
	Pyrimethamine	Inhibition of the biosynthesis of parasitic folate, interrupting nucleic acid synthesis and parasite replication
	+ Sulfadiazine	
	+/− ^5^ Steroids	
	Intravitreal Clindamycin	Inhibition of protein synthesis
	+ ^5^ Steroids	
	Trimethoprim	Inhibition of the biosynthesis of parasitic folate
	+ Sulfamethoxazole	Bacteriostatic sulfonamide interfering with folic acid synthesis
	+ ^2^ Steroids	
	Atovaquone	Targeting the mitochondrial electron transport and the mitochondrial cytochrome bc1 complex
	^4^ Azitromycin+/− Pyrimethamine	Inhibition of protein synthesis+/− biosynthesis of parasite folate
**Toxoplasmosis in immunocompromised patients**		
**(cycles of different doses in induction and maintenance therapy)**		
	^2^ Pyrimethamine	Inhibition of the biosynthesis of parasitic folate, interrupting nucleic acid synthesis and parasite replication
	+ ^2^ Sulfadiazine	Reduction of the harmful side effects
	+ Folinic acid (leucovorin)	
	^2^ Pyrimethamine	Inhibition of the biosynthesis of parasitic folate
	+ ^3^ Clindamycin	Inhibition of protein synthesis
	+ Folinic acid	Reduction of the harmful side effects
	^2^ Trimethoprim	Inhibition of the biosynthesis of parasitic folate
	+ Sulfamethoxazole	Bacteriostatic sulfonamide interfering with folic acid synthesis
	^2^ Pyrimethamine	Inhibition of the biosynthesis of parasitic folate
	+ Folinic acid	Reduction of the harmful side effects (i.e., bone marrow myelosuppression)
		Targeting the mitochondrial electron transport and the mitochondrial cytochrome bc1 complex
	+ Atovaquone	
	^2^ Sulfadiazine	Inhibition of the biosynthesis of parasitic folate
	+ Atovaquone	Targeting the mitochondrial electron transport and the mitochondrial cytochrome bc1 complex
	^2^ Pyrimethamine	Inhibition of the biosynthesis of parasitic folate
	+ Folinic acid	Reduction of the harmful side effects (i.e., bone marrow myelosuppression)
		Inhibition of protein synthesis
	+ ^4^ Azithromycin	

^1^ Side effects: Hematologic side effects (neutropenia, thrombocytopenia, and leukopenia, among others), in rare cases agranulocytosis, toxic epidermal necrolysis, hepatic necrosis, teratogenic potential if used during the first trimester of pregnancy. ^2^ Side effects: Hematologic side effects (neutropenia, thrombocytopenia, and leukopenia, among others), elevated liver enzymes, elevated creatinine levels. ^3^ Side effects: may cause diarrhea, and *Clostridium difficile* infection. ^4^ Side effects: may be associated with hearing problems. ^5^ Side effects: Steroids can have detrimental effects on vision (endophthalmitis) and vision loss if used without concomitant antimicrobial therapy.

**Table 2:** Summary of *Toxoplasma gondii* therapeutic targets and their corresponding drugs.

Parasite Targets				
Apicoplast	Micronemes	Rhoptries	Mitochondria	Nucleus
**Inhibitors of Fatty** **Acid synthesis:** -Clodinafop -Thiolactomycin -Triclosan	BKI targeting TgCDPK1: -BKI-1294 -BKI-1294 analogs: Compounds 24 and 32) -BKI-1748	Oxindoles	Targeting HSP60	**Topo-isomerase 2 inhibitors:** -Daunorubicin -Trovafloxacin -Enrofloxacin -Gatofloxacin
**Inhibitors of 2-Isoprenoid synthesis:** -Fosmidomycin	SP230	6-azaquinazolines	Atovaquone	**Topo-isomerase 1 inhibitors:** -Artemisinin -Artemisone -Artemiside -Artemether -Harmane -Harmine -Non-harmane
**Inhibitors of DNA gyrase:** -Quinolones -Fuoroquinolones -Ciprofloxacin -Trovafloxacin -Ofloxacin -Temafloxacin		Pyrazolopyridines	ELQ-271	**DNA-intercalating agents:** -Fluphenasine -Thioridazine -Trifluoperazine -Hycanton -Phleomycin -Mitomycin C
**Inhibitors of Protein synthesis:** -Clindamycin -Spiramycin -Azithromycin		Chemical scaffolds	ELQ-316	**Ribonucleotide reductase inhibitors:** -Thiosemicarbazones -Hydroxyurea
		Thiazolidinone derivatives	ELQ-400	**Oxidative DNA damage/DNA binding:** -Resveratrol -Valproic acid
			Naphtoquinones	

## Data Availability

Not applicable.
